# Co-transplantation of Human Fetal Mesenchymal and Hematopoietic Stem Cells in Type 1 Diabetic Mice Model

**DOI:** 10.3389/fendo.2019.00761

**Published:** 2019-11-06

**Authors:** Babak Arjmand, Parisa Goodarzi, Hamid Reza Aghayan, Moloud Payab, Fakher Rahim, Sepideh Alavi-Moghadam, Fereshteh Mohamadi-jahani, Bagher Larijani

**Affiliations:** ^1^Cell Therapy and Regenerative Medicine Research Center, Endocrinology and Metabolism Molecular-Cellular Sciences Institute, Tehran University of Medical Sciences, Tehran, Iran; ^2^Metabolomics and Genomics Research Center, Endocrinology and Metabolism Molecular-Cellular Sciences Institute, Tehran University of Medical Sciences, Tehran, Iran; ^3^Brain and Spinal Cord Injury Research Center, Neuroscience Institute, Tehran University of Medical Sciences, Tehran, Iran; ^4^Obesity and Eating Habits Research Center, Endocrinology and Metabolism Molecular-Cellular Sciences Institute, Tehran University of Medical Sciences, Tehran, Iran; ^5^Health Research Institute, Thalassemia and Hemoglobinopathies Research Center, Ahvaz Jundishapur University of Medical Sciences, Ahvaz, Iran; ^6^Endocrinology and Metabolism Research Center, Endocrinology and Metabolism Clinical Sciences Institute, Tehran University of Medical Sciences, Tehran, Iran

**Keywords:** cell transplantation, diabetes mellitus, fetal stem cells, hematopoietic stem cells, mesenchymal stromal cells, molecular imaging

## Abstract

**Introduction:** Cell therapy can overcome the limitation of conventional treatments (including different medications and β cell replacement) for type 1 diabetes. Based- on several studies human fetal mesenchymal and hematopoietic stem cells are ideal candidates for stem cell therapy. On the other hand, co-transplantation of them can improve their effects. Accordingly, the aim of this research is co-transplantation of human fetal mesenchymal and hematopoietic stem cells in type 1 diabetes.

**Materials and Methods:** The liver of legally aborted fetus was harvested. Then, mononuclear cells were isolated and extracted mesenchymal stromal cells and CD34^+^ hematopoietic stem cells were cultured. Expression of pluripotency markers were evaluated. For molecular imaging, mesenchymal stromal cells were labeled using GFP- vector. BALB/c inbred male mice were modeled by injection a single dose of Streptozotocin. Diabetic animals were received stem cells. After stem cell transplantation, *in vivo* imaging was performed and blood glucose levels were measured weekly.

**Results:** Fetal mesenchymal stromal cells were demonstrated differentiation potential. Expression of pluripotency markers were positive. The mean of blood glucose levels were reduced in mixed mesenchymal and hematopoietic stem cells transplantation. A lot of GFP-labeled mesenchymal stem cells were engrafted in the pancreas of animal models that received a mixed suspension of hematopoietic and mesenchymal stromal cells.

**Conclusions:** Human fetal stem cells are valuable source for cell therapy and co-transplantation of mesenchymal stromal cells can improve therapeutic effects of hematopoietic stem cells.

## Introduction

Diabetes mellitus (DM) is a metabolic disease with uncontrolled high blood glucose levels and severe secondary complications over time (1). In 2017, 451 million people around the world were estimated to have diabetes (which approximately half of them are undiagnosed). This number was expected to rise to 693 million in 2045 (2). Type 1 diabetes (T1D) is known as an autoimmune disease with the absence of immunologic tolerance against β cell antigens, resulting in an immune response against the pancreatic islets, insulitis, and destruction of pancreatic β cells (3). Suppression of these autoimmune processes can induce immune tolerance, reset immune system, and subsequently regenerate endogenous β cell in diabetic patients. Since the essential purpose of cell-based regenerative medicine approaches is the replacement and repair of damaged cells, tissues, and organs to achieve normal function (4), β cell replacement seems to be the most promising approach for T1D treatment (5). But, its application on a large scale is limited because of a shortage of qualified transplantable islet cells and considerable side effects of immunosuppressive therapy (6). Herein, differentiation of stem cells to insulin producing cells and regulated secretion in progenitor stem cells could offer a novel therapeutic approaches to overcome these obstacles. The most effective stem cell therapy for T1D treatment is considered the autologous hematopoietic stem cells (HSCs), as HSCs can produce significative amounts of insulin and release it in response to blood glucose levels to maintain euglycemia (7–9). Additionally, a large body of evidence have supported the effect of HSCs on the immune system reset and immune-modulation and also a promising role in cell therapy for autoimmune diseases through enhanced neo-vascularization (3, 10). On the other hand, it is demostrated that co-transplantation of MSCs can improve engraftment of HSCs and subsequently ameliorate clinical cell transplantation efficacy and patients' outcome (11, 12).

Mesenchymal stromal cells (MSCs) which have increasingly introduced as a valuable source for cellular therapy in recent years can be derived from adult and fetus tissues such as bone marrow (BM), adipose tissue, peripheral blood, dental pulp, breast milk, the lung, and the heart (13–15). Multi-potent MSCs with self-renewal, hypo-immunogenicity, and immune-modulatory properties paved the way for the development of allogeneic stem cell transplantation (16). In other words, the immunomodulatory propesrties of MSCs have recently become equally exciting for researchers to use them in treatment of several diseases such as diabetes (6). Further, it has been demonstrated that MSCs are capable of migration to damaged tissue and differentiation toward β cells. They also can reduce inflammation and improve healing and regeneration process in damaged sites (17). Due to the age-related reduction in the adult MSCs properties, fetal tissues derived stem cells from legally aborted fetuses has proposed as impressive alternative sources (18). On the other hand, in comparison with adult stem cells, fetal stem cells have some advantages including notable homing, engraftment, and differentiation potential, in addition to lower immunogenicity (19). Hence, human fetal MSCs and HSCs, sounds as an ideal candidate for clinical stem cell transplantation trials. They are supposed to hinder pancreatic β-cell destruction and contribute to β-cell regeneration by patient, to preserve residual β-cell mass, and reduce failure of islet transplantation (20–22). In this study co-transplantation of human fetal MSCs and HSCs in type 1 diabetic mice model followed by evaluation of their migration and engraftment using molecular imaging methods.

## Materials and Methods

### Ethical Considerations

This study was approved by the ethics committee of Endocrinology and Metabolism Research Institute of Tehran University of Medical Sciences, (EMRI) in accordance with Helsinki declaration and guideline of Iranian Ministry of health and medical education (Code: EC-00214). Moreover, all animal and human experiments were performed according to codes of ethics, declared by Iranian national committee on biomedical research ethics.

### Fetal Tissue Procurement, Liver Disruption, and Mononuclear Cells Isolation

To reduce the risk of bias, one therapeutically and legally aborted fetus at 6–12 weeks of gestational age was procured after obtaining written informed consent according to ethical committee approval and therapeutic abortion act (23). Moreover, a medical history was obtained and a complete physical examination was performed for the evaluation of mother (donor)'s eligibility as the fetus donor. In order to conduct serological tests for viral transmissible infectious diseases as well as rapid plasma reagin (RPR) test for syphilis, the blood samples were obtained from the fetus donors (mothers), at the abortion time (24).

The aborted fetus was transferred to the current good manufacturing practice (cGMP) facility in a sterile container and kept at approximately 4°C until the initiation of processing. Tissue processing was started as soon as possible (after abortion and total ischemic time under 36 h were included). All procedures of tissue and cell processing were performed in the cGMP-compliant stem cell manufacturing facility (clean room), under laminar air flow cabinet and in accordance with cGMP guidelines (24). One sample from the transfer medium were tested for aerobic and anaerobic bacteria and also fungi in order to evaluate any probable contaminations, before the processing. The fetus was weighed and washed with 5% povidone iodine solution once, followed by washing three times with sterile phosphate buffer saline (PBS) (CliniMACS®, Miltenyi Biotec, Germany). Then, the fetus was transferred on a sterile tray and fetal liver was harvested using a midline laparotomy and put in a 100 mm sterile petri dish. Adjacent tissues were eliminated and the liver tissue was minced into small pieces. Small pieces of liver were placed into a homogenizer and partially disrupted. In the following step, homogenized liver pieces were passed through the 70 μm cell strainer (Becton Dickinson, USA) adding PBS-EDTA (Ethylene Diamine Tetra Acetic Acid, 1 millimolar per 1 ml PBS). The filtered liver suspension was diluted with PBS-EDTA and then 7 ml of these solution was carefully transferred over 3 ml of Ficoll-Paque^TM^ PREMIUM (GE Healthcare Life Sciences, USA) in 15 mL falcon tubes (TPP, Switzerland). Then, it was centrifuged at 500 × g for 30 min at 22°C (Hettich 320R, Germany). The upper layer was aspirated and then the mononuclear cells (MNCs) layer was carefully collected. MNCs were transferred to a new 50 ml falcon tube (TPP, Switzerland) and were washed by the addition of PBS. Next, cells were centrifuged at 300 × g for 5 min at 22°C and the supernatant were removed. Finally, isolated pellet including MNCs re-suspended in 3 ml culture medium and were counted by NucleoCounter® NC-100TM (Chemometec, Denmark).

### Liver-Derived Mesenchymal Stromal Cells Expansion

Around 1 × 10^4^ MNCs per cm^2^ were seeded into the 75 cm^2^ filter cap flask (TPP, Switzerland) containing 12 ml Dulbecco's Modified Eagle Medium-low glucose (DMEM-LG) (PAA, Austria) supplemented with 15% pharma grade fetal bovine serum (FBS) (PAA, Austria). All flasks were incubated at 37°C, 5% CO_2_, and 98% humidity in CO_2_ incubator (Memmert INC108med, Germany). After 48 h, the culture medium was renewed and non-adherent cells and derbies were discard and fresh media were added to flasks. During cell culture period, the media were regularly renewed every 72 h until the adherent cells reach 80–85% confluency. After that, the adherent human fetal liver-derived MSCs (hFL-MSCs) were detached using TrypLE™ Express (Invitrogen, USA). Single cell suspension viability and purity were assessed using NucleoCounter® NC-100™. At 2nd or 3rd subcultures, hFL-MSCs were prepared for cryopreservation, karyotyping, and complementary characterization methods. Microbiological tests were performed before cell isolation, during, and also after cell expansion.

### Growth Kinetics

To demonstrate hFL-MSCs growth kinetics, growth curve was assessed to define lag, log (exponential), and plateau phases (25).

### Characterization of Liver-Derived Mesenchymal Stromal Cells

For characterization of hFL-MSCs (1) the cell surface markers (CD markers) were evaluated to detect the immunophenotypic characteristics of hFL-MSCs. (2) osteogenic, adipogenic, and chondrogenic differentiation potential of hFL-MSCs were assessed. (3) Cytogenetic stability of hFL-MSCs at 10th subculture was evaluated by karyotyping.

#### Flow Cytometric Analysis

Isolated cells were assessed for several MSCs markers using flow cytometry. Antibodies against CD11b, CD19, CD34, CD45, CD105, CD73, CD90, and HLA-DR; (all from Abcam, USA) were used in flow cytometry (26) and then labeled hFL-MSCs were analyzed on a fluorescence-activated cell sorting analysis (FACS; Partec). The cell cycle analysis was also performed by flow cytometry using specific staining buffer including PBS with RNase A (Sigma, USA), and Propidium Iodide (PI) (Sigma, USA).

#### Differentiation Potential

Multipotent MSCs are capable of differentiating toward adipocytes, osteocytes, and chondrocytes. The hfL-MSCs were differentiated using StemPro® Osteogenesis, Adipogenesis, and Chondrogenesis Differentiation Kits respectively (life technologies, USA) which is described below:

##### Adipogenic differentiation

The 3rd subculture of hfL-MSCs were suspended in adipogenesis medium in a four-well plate (Nunc, Thermo Scientific, USA) at a density of 2.5 × 10^4^ cells/well. Cells were incubated with the adipogenesis medium composed of 50 μg/ml indomethacin, 10^−7^ M dexamethasone, and 50 μg/ml ascorbate-sodium 2-phosphate. The culture medium was renewed every 3–4 days. After 14 days, cells were fixed with 4% paraformaldehyde and stained with Oil Red O (Sigma, USA) in order to stain lipid droplets in differentiated cells and counterstaining with hematoxylin were done.

##### Osteogenic differentiation

For osteogenic induction, hFL-MSCs suspension in osteogenic differentiation medium containing DMEM with 10 mM ß–glycerol phosphate, 50 μg/ml ascorbate sodium 2-phosphate, and 10^−7^ M dexamethasone were seeded to a 4-well cell culture plate at a density of 1.5 × 10^4^ cells/well. After 25 days of incubation and every 3 days medium exchanging, cells were fixed with 4% paraformaldehyde and calcium deposits were detected with Alizarin Red S (Sigma, USA) solution followed by hematoxylin staining.

##### Chondrogeinc differentiation

Chondrogenic induction was performed under micromass conditions in StemPro® chondrogenic differentiation medium composed of α-MEM supplemented with 10 ng/mL TGF-β1, 10 ng/ml TGF-β3 and 1% insulintransferrin-selenous acid premix according to the manufacturer's instructions. To this end, 3 × 10^5^ of hFL-MSCs suspension in 0.5 ml chondrogenic medium were pelleted after 5 min of centrifugation at 200 × g in a conical tube. The cell pellet and supernatant were incubated in tubes with loosened cap. The chondrogenic medium was carefully changed every 3 days and after 21 days the pellet was fixed with 4% paraformaldehyde. The cell pellet was then embedded in paraffin and sectioned to prepare slides. Then, cartilage formation was detected with Alcian Blue (Sigma, USA) staining.

#### Cytogenetic Evaluation

Cytogenetic stability of hFL-MSCs at 10th subculture was evaluated using conventional G-banding karyotyping. Briefly, at 70% confluency, hFL-MSCs were incubated with 0.1 μg/mL colcemid (Gibco-Invitrogen) for 2–3 h in 5% CO_2_ and 37°C. Then 3:1 methanol and acetic acid were added for cell fixation. Then, cells were spread on the lamella surface and chromosome profile, structural abnormalities, chromosomal gains, and deletions were evaluated.

### Reverse Transcriptase Polymerase Chain Reaction

To appraise the pluripotency capacity of hFL-MSCs, reverse transcription-polymerase chain reaction (RT-PCR) was performed using pluripotency marker genes including OCT4, NANOG, SOX2, and REX1. Table 1 shows target gene primers's sequences and their product sizes. Briefly, total RNA was extracted from the hF-MSCs using the RNeasy Mini RNA isolation kit (Qiagen, Germany). The cDNA synthesis kit was applied to produce cDNA from total RNA (Qiagen, Germany). RT-PCR was performed by a master mix including RNase-free water, RT-PCR Buffer, dNTP Mix, primers, RT-PCR Enzyme Mix, and RNase inhibitor. Amplified PCR products were separated on a 2% agarose gel electrophoresis containing SyberSafe (Invitrogen, USA).

**Table 1:** Primers used for RT-PCR.

**Target gene**	**Primer sequence**	**Product size (bp)**
NANOG	F 5′ AAAGTCTTAAAGCTGCCTTAAC 3′ R 5′ CAGTCGGATGCTTCAAAG 3′	130
REX1	F 5′ TTTACGTTTGGGAGGAGG 3′ R 5′GTGGTCAGCTATTCAGGAG 3′	150
SOX2	F 5′GGGAAATGGAAGGGGTGCAAAAGAGG 3′ R 5′GGGGCTTCTGCATACTCAAA 3′	151
OCT4	F 5 ′GTTCTTCATTCACTAAGGAAGG 3′ R 5′CAAGAGCATCATTGAACTTCAC 3′	101
GAPDH	F 5′GTTCTTCATTCACTAAGGAAGG 3′ R 5′ CAAGAGCATCATTGAACTTCAC 3′	122

### GFP Labeling of hFL-MSCs

Cultured 70–80% confluent hFL-MSCs were exposed to green fluorescent protein (GFP)-encoding lentiviral vector (pLVIRES-GFP). The cells were transduced with pLVIRES-GFP at the multiplicity of infection in the presence of 5 mg/ml polybrene and the second transduction was repeated after 48 h. Subsequently, transduced cells were evaluated for expression of GFP using inverted fluorescent microscope (Nikon, Japan) (27).

### Hematopoietic Colony Forming Assay

StemMACS HSC-CFU Media (Miltenyi Biotec, Germany) was thawed overnight at 4°C. After thawing, the medium was vigorously shacked and left for 10–20 min to allow air bubbles to rise to the top. Hematopoietic colony-forming assay was performed MNCs, from fetal liver that were isolated by density gradient. According to the manufacturer's instructions, around 1 × 10^5^ fetal liver MNCs in 0.3 ml Iscove's Modified Dulbecco's Medium (IMDM) supplemented with 2% FBS were immediately added to a 3 ml StemMACS HSC-CFU media prior to plating. Then, the suspension was vigorously shacked until the cells were well-suspended. After rising air bubbles, 1.1 ml of the cell/methylcellulose suspension was aliquot into each of two 35 mm petri dishes. Then, the dishes were gently rotated and pairs of 35 mm dishes placed in a 100 mm dish adding a third 35 mm dish containing 3 ml sterile water to the 100 mm dish without lid in order to maintain an adequately mummified atmosphere during culturing. The dishes were incubated for 14–16 days in a humidified incubator at 37°C and 5% CO_2_. Based on StemMACS HSC-CFU assay data sheet, hematopoietic colonies were classified by color and morphology using an inverted microscope and comparing them with the reference photos provided by the manufacturer (28).

### Fetal HSCs Isolation and Expansion

Human fetal MNCs were isolated by density gradient by Ficoll-Paque™, and cell pellet re-suspended in the buffer for the following labeling and separation procedures. To prevent capping of antibodies on the cell surface and non-specific cell labeling, MNCs were kept cold, and pre-cooled solutions were used. CD34^+^ hematopoietic stem cells were isolated by CD34 MicroBead Kit UltraPure and SuperMACS II (Miltenyi biotec, Germany) based on manufacturer's instructions. For optimal performance, cells were passed through 30 μm nylon mesh to remove cell clumps and provide a single cell suspension. Prepared cells were re-suspended in 300 μl of buffer (for up to 108 total cells) and 100 μl of FcR blocking reagent was added. Subsequently, 100 μl of CD34 Micro Beads UltraPure was added, and mixed and was incubated for 30 min in the refrigerator (2–8°C). The next step was washing process with buffer and centrifuging at 300 × g for 10 min. After that the supernatant was completely discarded and cells were re-suspended in 500 μl of the buffer. LS column and SuperMACS II were used for up to 1 × 10^8^ labeled cells according to the manufacturer's instructions. LS column placed in the magnetic field of the SuperMACS II. Column prepared by rinsing with the 3 ml of buffer and then cell suspension was applied onto the column carefully and was collected. After that, column was washed with the buffer and unlabeled cells collected. At the following steps, the column was removed from the separator and placed on a collection tube and washed with appropriate amount of buffer. All steps were repeated using new column. Approximately 5 × 10^3^ isolated CD34^+^ HSCs/ml were seeded in StemMACS HSC expansion medium XF (Miltenyi biotec, Germany) supplemented with StemMACS HSC expansion cocktail and incubate at 5% CO2 and 37°C. Expanded cells were counted and released for transplantation in animal models.

### Characterization of Hematopoietic Stem Cells

Isolated HSCs were characterized by flow cytometry based on CD11a, CD18, CD34, CD44, CD45 markers. In this context, mono-color flow cytometry was carried out within 12 h post- isolation and cells were incubated with monoclonal antibodies conjugated with phycoerythrin (1:100; BD Bioscience, Germany). Finally, fluorescence intensity was quantified using flow cytometer (FACSCalibur; BD Biosciences, USA) and data were analyzed by FlowJo software.

### Adipose Tissue-Derived Stromal Cells Isolation and Expansion

Human Adipose tissue-derived stromal cells (ASCs) were isolated and cultured as we described previously (29). Adipose tissue was aseptically harvested after obtaining written informed consent from donors. Adipose tissues were washed twice with PBS and then minced into small pieces and enzymatically digested using collagenase-NB6 (GMP-grade, Serva Electrophoresis GmbH, Germany). Mature adipocytes, debris, and liquid portion were discarded and the stromal vascular fraction (SVF) was re-suspended in PBS and filtered through a nylon mesh. Subsequently, this suspension was transferred into culture flasks and incubated at 37°C, 5% CO_2_ and humidified air. At 3rd subculture, cell characterization (flow cytometry, differentiation potential) were performed.

### Animal Modeling

As described previously, animal experiments were performed according to the ethical guideline on animal research, declared by Iranian national committee on biomedical research ethics. Furthermore, current study was conducted according to National Institutes of Health (NIH) guidelines for laboratory animals' care and handling and we tried to minimize animal suffering and to reduce the number of animals used in this project (based on 3 R's rule). Healthy BALB/c inbred male mice [7-12-week old](mean ≈ 10 week old), [20-30 g] (mean ≈ 24 g) were purchased from the Pasteur institute (Tehran, Iran) and housed in individual cages at humidified constant temperature (22±1°C) with a 12 h light–dark cycle, and were allowed free access to food and tap water. Then, animals were divided into four groups and (the number of animals in each group were defined according to similar previous studies) (30). Four groups were as follows:

- Group 1 (*n* = 5); a mixed suspension of 2 × 10^5^ HSCs and 1 × 10^5^ MSCs were transplanted via tail vein.
- Group 2 (*n* = 4); a mixed suspension of 2 × 10^5^ HSCs and 1 × 10^5^ MSCs were intra-pancreatically transplanted.
- Group 3 (*n* = 4); 2 × 10^5^ MSCs were transplanted via tail vein.
- Group 4 (*n* = 4); diabetic controls with PBS infused via tail vein.

A single dose 180 mg/kg Streptozotocin (STZ) in sodium citrate buffer (PH 4.5) was intraperitoneally injected in order to induce diabetes in mice. During the early stages of low blood sugar, mice were fed with dextrose water. Blood samples were taken from the tail vein and blood glucose levels were frequently monitored with a glucometer (GLUCOCARD^TM^ 01, Japan) under non-fasting conditions. Due to the fact that previous reports defined diabetes as non-fasting blood glucose levels more than 250 or 300 mg/dl (31), in this study mice with non-fasting blood glucose levels more than 300 mg/dl were considered as diabetic models.

### Stem Cell Transplantation

**Figure 1:**
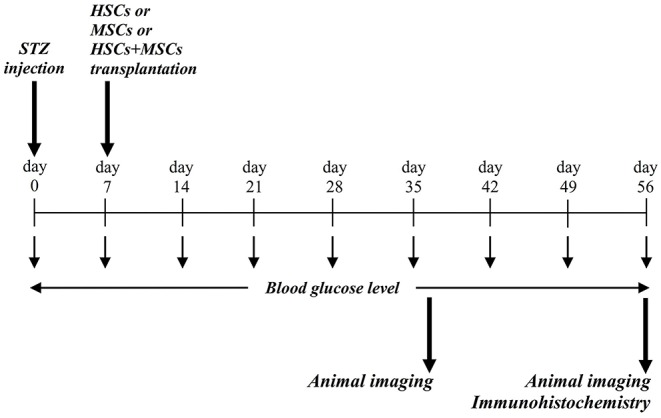
Conceptual framework and experimental design. STZ was injected via intra-peritoneal route, and blood glucose level were assessed during the study period. At day 37 and 56, animals were evaluated for probable stem cell homing and engraftment using *in vivo* imaging.

Diabetic mice were anesthetized with an intra-peritoneal injection of a mixture of ketamine and xylazine, 45 and 8 mg/kg, respectively. According to pervious investigations (8, 10, 12) each group received its predefined specific stem cells as follows: group 1: a mixed suspension of 2 × 10^5^ HSCs and 1 × 10^5^ GFP-labeled MSCs via tail vein concurrently, group 2: a mixed suspension of 2 × 10^5^ HSCs and 1 × 10^5^ GFP-labeled MSCs by intra-pancreatic injection, group 3: 2 × 10^5^ GFP-labeled MSCs via tail vein, and group 4 or control group: PBS via tail vein. Figure 1 summarizes the framework of the experimental phase including STZ injection, stem cell transplantation, blood glucose monitoring, and evaluating stem cell homing and engraftment using imaging methods. In intravenous injection technique, diabetic mice (group 1 and 2) were anesthetized and then cell suspensions were slowly injected via tail vein. In Intra-pancreatic technique, animals were anesthetized and using a left posterior incision the pancreas was totally exposed. Subsequently, a mixed suspension of 2 × 10^5^ HSCs and 1 × 10^5^ GFP-labeled MSCs were directly injected in different anatomic parts of the pancreas of diabetic mice.

### Molecular Imaging

At different time points (1 and 2 months) after stem cell transplantation, animals were anesthetized and carefully shaved in order to *in vivo* imaging to evaluate stem cells homing and engraftment using Kodak *In Vivo* Imaging Systems (Kodak, USA). Moreover, in the second month after transplantation lungs, liver, pancreas, and spleen of mice were harvested and stem cell homing was tracked using above mentioned imaging system.

### Statistical Analysis

Statistical analysis were performed using SPSS 19.0.A two-way repeated measures analysis of variance (ANOVA) was applied for the comparison of blood glucose levels. Blood glucose level was expressed as mean ± standard error of mean. A significant difference was designated as *p* < 0.05.

## Results

### Morphology and Immunophenotypical Characteristics of MSCs

hFL-MSCs and adult ASCs isolated from healthy donors were morphologically evaluated at 3rd and 4th subcultures, respectively. Both types of MSCs demonstrated the same spindle-shaped fibroblast-like morphology. At the same microscopic magnitude, hFL-MSCs were smaller than adult ASCs.

### Growth Kinetics

Growth kinetics of hFL-MSCs were evaluated at 3rd subculture. Growth curves showed an initial lag phase approximately 3 days, followed by a log or exponential phase for about 3 days and finally the plateau phase was observed after 7 days.

### Characterization of Human Fetal Liver Derived Mesenchymal Stem Cells

#### Immunophenotyping

**Figure 2:**
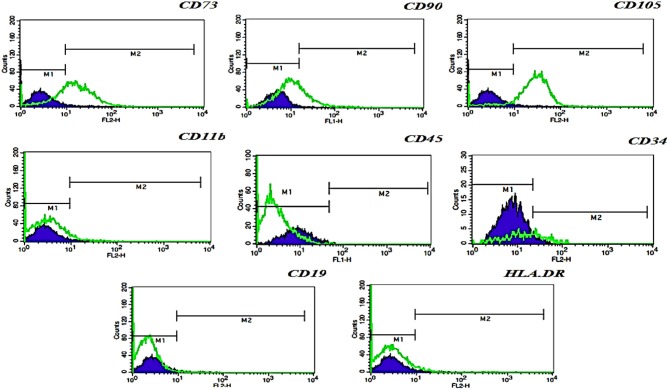
Flow cytometric analysis of hFL-MSCs marker expression. Histograms indicates the immunophenotype of hFL-MSCs which were positive for CD73, CD90, CD105, but negative for CD11b, CD45, CD34, CD19, and HLA-DR. In hFL-MSCs established MSCs surface markers including CD73, CD90, and CD105 were highly expressed (>95%), whereas hematopoietic stem cells markers like CD11b, CD19, CD34, CD45 and also HLA-DR, did not express (>98%).

The immunophenotype of hFL-MSCs isolated from the first trimester was determined by flow cytometry (Figure 2). In hFL-MSCs established MSCs surface markers including CD73, CD90, and CD105 were highly expressed (>95%), whereas hematopoietic stem cells markers like CD11b, CD19, CD34, CD45 and also HLA-DR, did not express (>98%).

#### Cell Cycle Analysis

**Figure 3:**
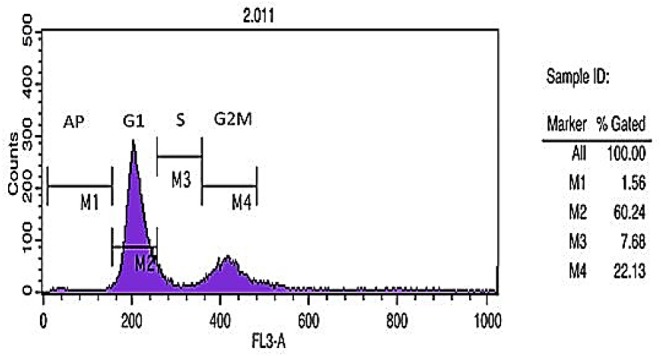
Cell cycle analysis. DNA content histogram depicts apoptotic cells (Ap.), cells in G1, S, and G2/M phases of the cycle.

DNA content histogram was prepared using flow cytometric analysis of cell cycle DNA staining method. Different cell cycle phases including G1, S, and G2 demonstrated the percent of apoptotic cells (1.56%), 2n cells in G1 phase (60.24%), DNA synthesis or S phase (7.68%), and 4n cells in G2M (mitosis) phase (22.13%) (Figure 3).

#### Differentiation Potential

**Figure 4:**
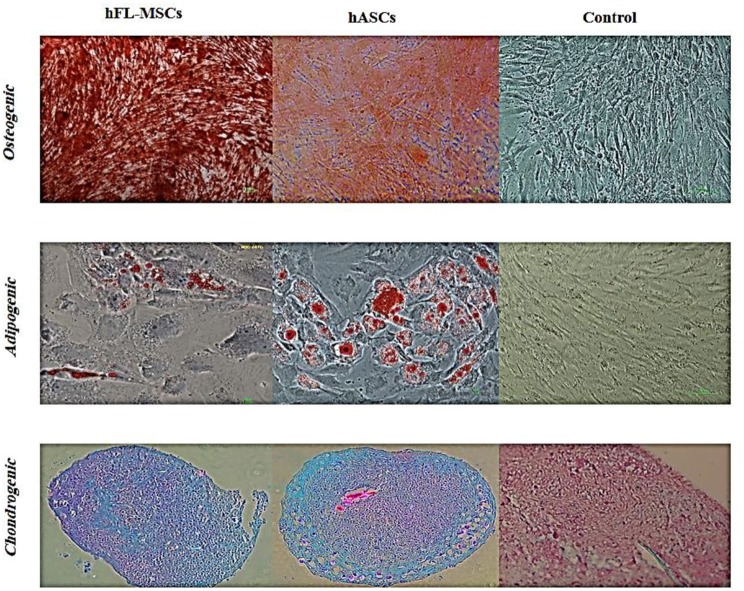
Differentiation potential of hFL-MSCs and ASCs. Human fetal mesenchymal and also adult adipose tissue stromal cells were induced by differentiation medium. Lipid droplets of adipocytes, calcium deposits of osteocytes, and cartilage formation in chondrocytes were visualized by Oil-Red-O, Alizarin Red S, and Alcian blue staining, respectively.

In order to evaluate differentiation potential of cultured hFL-MSCs and adult ASCs, their potency was performed to produce mesodermal lineages, osteogenic, adipogenic, chondrogenic, as a well-known multipotency property of MSCs. Both hFL-MSCs and adult ASCs were successfully differentiated into adipocytes (Oil Red O staining), osteocytes (Alizarin Red S staining), and chondrocytes (Alcian blue staining) when cultured in adipogenic, osteogenic and chondrogenic media (Figure 4).

#### Cytogenetic Analysis

At 10th subculture, cytogenetic analysis was performed to evaluate chromosomal stability of hFL-MSCs using G-banding karyotyping technique that showed a normal male karyotype 46, XY with no evidence for structural and numerical chromosomal aberrations.

### Polymerase Chain Reaction

**Figure 5:**
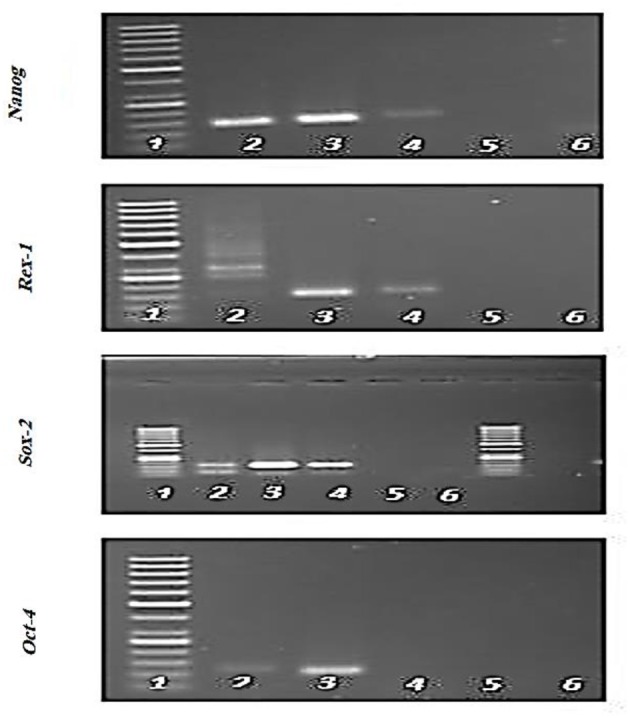
Expression of specific pluripotency markers in hFL-MSCs. Expression of Nanog, Sox-2, and Rex-1 and lack of expression of Oct-4 was demonstrated by RT-PCR in hFL-MSCs. 1: DNA ladder; 4: hFL-MSCs.

Expression of specific pluripotency markers (Figure 5) including Oct-4, Nanog, Sox-2, and Rex-1 in hFL-MSCs were determined by reverse transcription-polymerase chain reaction RT-PCR. As it can be seen in Figure 5, the mRNA expression of Nanog, Sox-2, and Rex-1 was positive while, the expression of Oct-4 was negative in hFL-MSCs.

### Colony Forming Potential in Human Fetal Hematopoietic Stem Cells

Human fetal HSCs colonies demonstrated colony-forming unit-granulocyte, macrophage (CFU-GM) pattern, including colony-forming unit-granulocyte (CFU-G), or colony-forming unit-macrophage (CFU-M), or both cell types (CFU-GM). CFU-GM depicted as multiple cell clusters with dense cores. The monocyte lineage cells were large cells with an oval to round shape with a grainy and gray center. The granulocyte lineage cells were smaller and also more uniform than macrophage cells with a round and bright feature. Colony-forming unit-granulocyte, erythroid, macrophage, megakaryocyte (CFU-GEMM) was showed large colonies (more than 500 cells) containing erythroblasts and at least two other lineages after 16 days. CFU-GEMM had a highly dense core with an indistinct border between the core and peripheral cells.

### Flow Cytometry Analyzing of Hematopoietic Stem Cells

Fetal HSCs characterization were determined by flow cytometry (Table 2). In human fetal HSCs surface markers including CD44, CD45, and CD34 were highly expressed (>20%), and the lack of expression of non-hematopoietic stem cells markers like CD11a and CD18 was observed.

**Table 2:** Mean fluorescence intensity of human fetal derived hematopoietic stem cells.

**CD marker**	**Mean fluorescence intensity ± SE**
CD11a	3.53 ± 0.04
CD18	3.60 ± 0.03
CD44	46.60 ± 0.1
CD45	20.06 ± 0.04
CD34	21.2 ± 0.1

### Expression of GFP in hFL-MSCs

The expression of GFP after GFP gene transfection in hFL-MSCs at 3rd subculture. Forty-eighty hours after GFP gene transfection, the hFL-MSCs were expressed GFP which was observed as green and spindle-shaped cells under fluorescence microscope (Olympus, Japan).

### Blood Glucose Levels Following Stem Cell Transplantation

**Figure 6:**
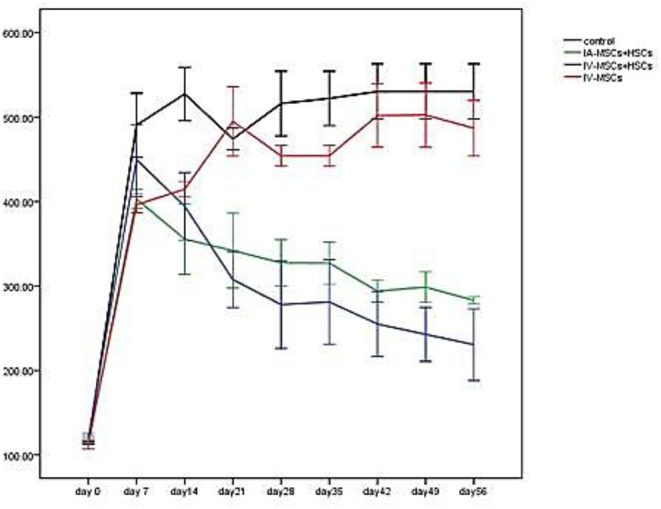
The mean of blood glucose levels before and after stem cell transplantation in different study groups (mean ± SEM).

Five to seven days following STZ administration, hyperglycemic mice (blood glucose > 350 mg/dl for two consecutive times) were considered as diabetic one which demonstrated an average blood glucose level more than 4,305 mg/dl (435.82 ± 57.17). Blood glucose levels of STZ induced mice were monitored and documented weekly from day 0 (STZ injection) till the 56th day using animal imaging. Table 3 demonstrates the blood glucose levels of mice in different groups during study period. In comparison with group 3 (*n* = 4) which received MSCs intravenously and group 4 (*n* = 4) as control group that did not undergo stem cell treatment, 2 weeks after STZ injection, coincident with 1 week following stem cell transplantation, the average of blood glucose level of groups 1 (*n* = 5) and 2 (*n* = 4) [were significantly reduced (groups 1 and 2 were received mixed MSCs and HSCs intravenously and intrapancreatically, respectively and in each group one mouse was died because of high blood sugar in the first days] (Figure 6). Accordingly, 7 weeks following stem cell transplantation, the average of blood glucose levels of groups 1 and 2 mice were significantly lower than that of groups 3 and 4. In addition, no significant differences were detected between group 1 and 2, and also between group 3 and 4 during blood glucose level monitoring of diabetic mice.

**Table 3:** Blood glucose levels in different groups during study period.

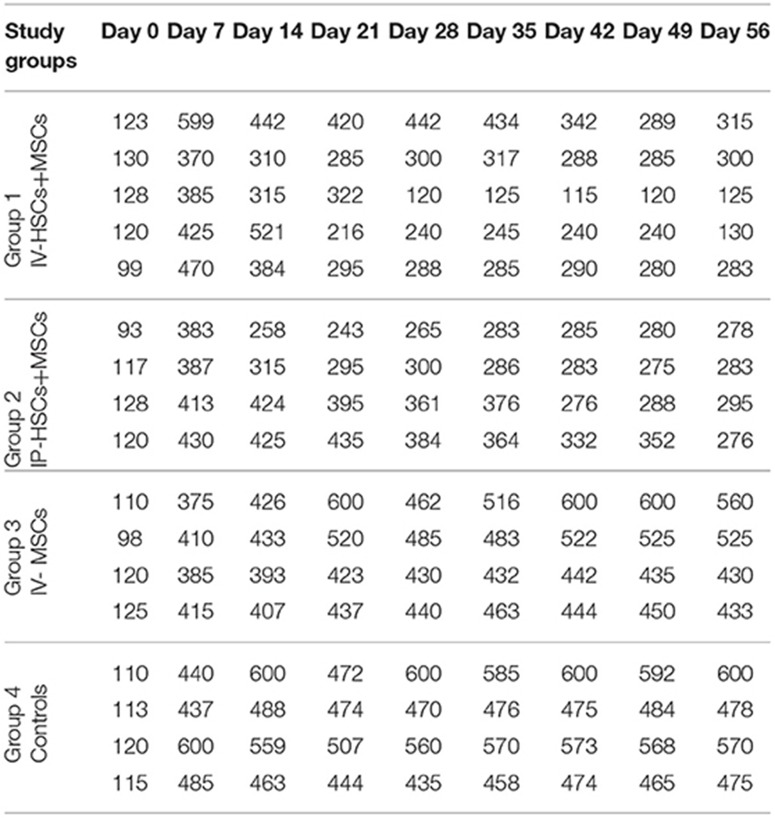

### Molecular Imaging

#### *In vivo* Imaging

**Figure 7:**
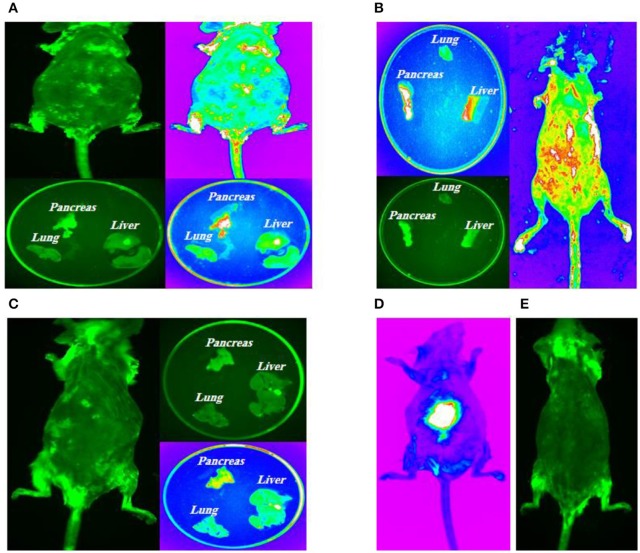
Intra**-**pancreatically co-transplantation of a mix cell suspension (HSCs and GFP-labeled MSCs). **(A)** Left posterior approach to mouse pancreas; **(B)** Spleen as an anatomical guide to expose pancreas; **(C)** Kidney, spleen and pancreas are totally exposed and stem cells are directly transplanted into all parts of pancreas (head, body, and tail); **(D)** Positive control model; **(E)** Negative control model.

10 days, 1 month, and 2 months following cell transplantation, molecular imaging were done in order to track transplanted stem cells using Kodak small animal imaging system. The results of *in vivo* molecular imaging have demonstrated different patterns of stem cell homing as shown in Figures 7A–D. These patterns were depended on types of transplanted cells and administration routes. In brief, in group 1 (Figure 7A) fluorescent signals were seen in thoracic area (lungs) and also in the upper segment of the abdomen (liver and pancreas). In group 2 (Figure 7B) fluorescent signals were observed in the abdominal area (pancreas) with no signals in the thoracic area (lungs). In group 3 (Figure 7C) some weak signals in the upper part of abdomen (pancreas) were detected and in group 4 (Figure 7E) as diabetic controls no signals were exhibited.

#### Imaging of Target Tissues

All mice in four groups were sacrificed after the experiment, and their tissues were harvested for molecular imaging. The results of fluorescent imaging of tissues is shown in Figure 7. In group 1, intravenously injected MSCs and HSCs, the highest signal density was detected in the pancreas which was followed by lungs (Figure 7A). Group 2, intra-pancreatically injected MSCs and HSCs, has demonstrated the highest signal density in the pancreas, while no signals was observed in lungs (Figure 7B). Finally, in group 3, intravenously injected MSCs, a weak signal density was detected in the pancreas with no signals in lungs and the liver (Figure 7C).

## Discussion

Cellular therapy as a fast-growing area and one types of advanced therapies tries to replace damaged and un-functional cells with the healthy and functional one (1, 32). In this respect, MSCs are considered as the most common stem cell source which has been used in various stem cell researches and cellular therapy trials. They can be isolated from different adult tissues and fetal tissues (33). In recent years, fetal stem cells (MSCs and HSCs) have been introduced as a valuable and attractive source for regenerative medicine because of several advantages over their embryonic and adult counterparts (34). In our study, morphology and immunophenotypic properties of fetal MSCs were similar to adult MSCs based on the minimal criteria for defining multipotent MSCs (26). Like to other studies, we demonstrated a stable karyotype at 10th subculture of human fetal stem cells. Growth curve of hFL-MSCs was drawn at 3rd subculture. It depicted an initial lag phase (around 3rd day) followed by a log (exponential) phase. Finally we had a plateau phase. Lag phase was happened immediately after re-expansion. In this study, the lag period lasted 2–3 days similar to other reports (25, 35). Lag phase allows the cells to recover from detachment and harvesting, repair the cytoskeleton, and restart the cell cycle. After the lag phase, log or exponential phase was started and the cell population was doubled in a determinable period (doubling time). In the next phase (the plateau or stationary phase), the growth fraction dropped to zero because of a high cell density and exhaustion of growth factors. Cell cycle analysis was performed using flow cytometry and revealed that, most of the hFL-MSCs were in G1 phase (60.24%) as normal diploid 2n cells. Also 22.13% of the cells were in G2M phase (4n) and meiotic state. While apoptotic cells were <2% and others (7.68%) were in S phase. Some previous studies demonstrated the existence of pluripotent subpopulations in human fetal tissues especially in first trimester (34). In this study we showed that hFL-MSCs can express some of the pluripotency markers like to ESs. Accordingly, our cells expressed Nanog, Rex-1, and Sox-2 but they did not express Oct-4 while, some previous researches showed that human fetal stem cells can express Oct-4 in addition to other pluripotent markers (e.g., Nanog, Sox-2, Rex-1, SSEA-3&4, Tra-1-61, Tra-1-81, etc.) (34). Expression of pluripotency markers is a crucial factor in differentiating into the three germ layers. All of these markers have important roles in pluripotency properties of fetal stem cells. For instance, Oct-4 inhibits stem cells differentiation, Nanog as a homeodomain protein guides pluripotency and differentiation of undifferentiated fetal and embryonic stem cells. In addition, fetal stem cells have also immunological advantages in comparison with adult stem cells. There is a lack of MHC class II (HLADP, HLA-DQ, and HLA-DR) and a lack or very low expression of MHC class I (HLA-A, HLA-B, and HLA-C) in fetal stem cells which can provide an immune tolerance advantage and a potential for both autologous and allogeneic cell transplantation (16). On the other hand, adult and especially fetal MSCs have demonstrated strong immunomodulatory effects as well. Therefore, fetal MSCs not only fail to induce allogeneic and xenogeneic immune response, but also strongly inhibit lymphocyte proliferation *in vitro* and in *vivo* (16). According to these properties of fetal stem cells and also immunomodulatory effects of MScs, we decided to perform co-transplantation of HSCs and MSCs from human fetal tissues in animal models of T1D without immunosuppression regimen to enhance HSCs engraftment and therapeutic effects of both types of cells. This concept was supported by several basic and clinical experiences in HSCs transplantation, co-transplantation of HSCs and MSCs (36), and also xenotransplantation of human fetal stem cells into animal models with or without immunosuppression (37). In the present study, 1 week following stem cell transplantation, mean blood glucose level were significantly reduced in group 1 and group 2 (received mixed MSCs and HSCs intravenously and intrapancreatically) in comparison with group 3 (MSCs intravenously) and control group (*P* value < 0.05). While, there were no significant differences between group 1 and 2, and also between group 3 and 4. These results indicate that co transplantation of MSCs and HSCs would be more effective in treatment of T1D and other incurable diseases. Also, based on our results and previous studies, MSCs can enhance engraftment and therapeutic effects of HSCs in animal models. In our study as a xenotransplantation setting, immunomodulatory effects of MSCs can be concluded from the results that indicates the advantages of co-transplantation manner in comparison with transplantation of only MSCs or only HSCs in animal models without immunosuppression regimen. Although, there are some studies that demonstrated the therapeutic effects of MSCs transplantation in rodent models of T1D (31), the reports elucidated more successful projects using co-transplantation of MSCs and HSCs (38). Different route of administration were suggested by investigators such as intravenously, intraperitoneally, intraarterially administration or injection in the site of injury (39). Fortunately, all of these methods were effective in treatment of T1D (40, 41). Like to Katuchova's study, we injected a mixed suspension of MSCs and HSCs in different parts of pancreas in diabetic mouse model (head, body, tail) using a posterolateral surgical incision. In our study no significant therapeutic differences were seen between intrapancreatic and intravenous injection of stem cells. While, co-transplantation of HSCs and MSCs showed significant therapeutic advantage over injection of single type of stem cell (MSCs). Some researchers elucidated that hFL-MSCs can enhance engraftment and maybe homing of other types of transplanted cells in animal models (42). We also elucidated that GFP-labeled MSCs migrated and engrafted into the site of injury (Pancreas).Furthermore, it has been demonstrated that most of the cells were entrapped in the lungs when they were administered intravenously. Of course, they could be able to redistribute in the body, but in short term this phenomenon significantly reduces the number of transplanted cells that received to the site of injury. Therefore, to overcome this problem, researchers suggested other routes such as intra-arterial, intrapancreatic, intrasplenic, etc. Accordingly, we compared the effect of intravenous and intrapancreatic route of injection and we did not find significant differences between their therapeutic effects but *in vivo* animal imaging showed that in group 1 (MScs and HSCs intravenously), the highest signal density was showed in the pancreas which followed by the lungs and group 2 (MSCs and HSCs intrapancreatically), demonstrated the highest signal density in the pancreas while no signals were seen in the lungs. Also it was elucidated that signal density in pancreas is higher in group 2 than group 3. There were some limitations in this project. Animal modeling according to the ethical issues was one of the limitations. Only, some animals successfully mimicked T1D following STZ injection. Therefore, we needed to use more animals to provide our sample size in each group. Also it would be better if researchers use an appropriate method for labeling of HSCs in addition to MSCs to show the engraftment and migration pattern of both types of cells following transplantation. On the other hand immunomodulatory and protective effects of MSCs can be tested. Moreover, it is advisable to include additional studies in control groups based on the route of injection and the type of cells along with additional reviews of entire groups to check the immunohistology, serum insulin levels, islet histology, and etc. Finally, we suggest that to achieve more efficiency, re- transplantation of stem cells in well-defined time intervals is necessary for *in vivo* and clinical stem cell transplantation projects.

## Conclusion

We concluded that human fetal stem cells especially from the first-trimester represent a developmentally less mature stem cells that express pluripotency markers similar to ESCs but without tumorigenicity. These properties and other advantages have introduced these cells as an invaluable source for cellular therapies. It is strongly suggested that co-transplantation of MSCs and fetal HSCs enhance HSCs engraftment and subsequently their therapeutic effects in various diseases including T1D. Our results revealed that, co-transplantation of MSCs and HSCs has more therapeutic effects on T1D in comparison with MSCs transplantation. Also it was elucidated that, following cell transplantation, most of cells engrafted in the site of injury such as pancreas in T1D.

## Data Availability Statement

This manuscript contains previously unpublished data. The name of the repository and accession number(s) are not available.

## Ethics Statement

The animal study was reviewed and approved by Ethics Committee of Endocrinology and Metabolism Research Institute of Tehran University of Medical Sciences (Code: EC-00214).

## Author Contributions

BA carried out most of the data acquisition and analysis, helped with drafting the article and approved the final version. PG accomplished the cell transplantation and statistical analysis. HA helped with data acquisition. MP and FR contributed to the study design and provided samples and animal models. SA-M helped with processing and interpretation of PCR and flow cytometry data. FM assisted in stem cell culture and characterization. BL supervised the project from the scientific view of point and advised on experimental design.

### Conflict of Interest

The authors declare that the research was conducted in the absence of any commercial or financial relationships that could be construed as a potential conflict of interest.

## Abbreviations

DM: Diabtes Mellitus
T1D: Type1 Diabetes
HSCs: Hematopoietic Stem Cells
MSCs: Mesenchymal Stromal Cells
BM: Bone Marrow
EMRI: Endocrinology and Metabolism Research Institute
RPR: Rapid Plasma Reagin
cGMP: current Good Manufacturing Practice
PBS: Phosphate Buffer Saline
DMEM-LG: Dulbecco's Modified Eagle Medium-low glucose
FBS: Fetal Bovine Serum
hFL-MSCs: human Fetal Liver-derived Mesenchymal Stromal Cells
RT-PCR: Reverse Transcription-Polymerase Chain Reaction
GFP: Green Fluorescent Protein
IMDM: Iscove's Modified Dulbecco's Medium
NIH: National Institutes of Health
SVF: Stromal Vascular Fraction
STZ: Streptozotocin.
